# Suspension fixation of iliac bone grafts under arthroscopy is an effective method for the treatment of unstable bony Bankart disease of the shoulder joint in patients with joint relaxation

**DOI:** 10.1007/s00167-022-07127-8

**Published:** 2022-08-30

**Authors:** Peng Zhou, HongBin Shao, MaoSheng Zhao, XiaoJie Yang, Zuobin Hao, Zhao Chen, Shensong Li, Peng Zhang

**Affiliations:** 1 Department of Sports Medicine, The 940th Hospital of Joint Logistic Support Force of Chinese People’s Liberation Army, Lanzhou, 730050 Gansu China; 2 Department of Sports Medicine, Huashan Hospital, Fudan University, Shanghai, 200040 China; 3 Department of Hand Surgery, The Third People’s Hospital of Jinan, Jinan, 250000 Shandong China; 4 The Third Department of Surgery, Qinghai Province Crops Hospital of Chinese People’s Armed Police Forces, Xi’ning, 810000 China

**Keywords:** Arthroscopy, Bone graft, Recurrent shoulder dislocation, Remplissage procedure

## Abstract

**Purpose:**

To evaluate the results of arthroscopic autologous iliac bone graft suspension fixation combined with the Remplissage procedure in the treatment of recurrent shoulder dislocation with bony Bankart lesions and joint hyperlaxity.

**Methods:**

From 2018 to 2020, 22 patients with joint laxity underwent arthroscopic autologous iliac bone graft suspension fixation and Bankart repair combined with the Remplissage procedure due to recurrent shoulder dislocation. Clinical assessment included range of motion (forward flexion, abduction, 90° external rotation, conventional external rotation, adduction, and internal rotation), visual analog scale (VAS) score, Rowe score, University of California Los Angeles (UCLA) score, and Western Ontario Shoulder Instability Index (WOSI) score. Post-operatively, the healing of the bone graft was evaluated with computed tomography (CT) scanning.

**Results:**

All 22 patients were followed up for a mean of 19.3 ± 4.1 months. CT imaging showed that the healing time of the bone graft was 6–8 weeks. The patient satisfaction rate was 100%, there were no cases of redislocation, all patients returned to their preinjury training state, and the fear test was negative. At the final follow-up, the UCLA, VAS, Rowe, and WOSI scores were 29.8 ± 2.1, 2.2 ± 0.8, 89.4 ± 4.2, and 482.3 ± 46.2, respectively (*p* < 0.001).

**Conclusion:**

Arthroscopic autologous iliac bone graft suspension fixation and Bankart repair combined with the Remplissage procedure are effective in preventing recurrent instability with joint hyperlaxity. Furthermore, no patient had redislocation.

**Level of evidence:**

IV.

**Supplementary Information:**

The online version contains supplementary material available at 10.1007/s00167-022-07127-8.

## Introduction

Recurrent anterior instability of the shoulder is a complex disorder in the clinical practice of sports trauma and orthopedics. According to a previous report, the prevalence of anterior glenohumeral instability is approximately 2% [14]. Recurrent dislocation can cause bone defects of the adjacent anteroinferior glenoid, forming bony Bankart lesions, and most patients also have Hill–Sachs injury [4, 29].

Unlike normal patients, those with joint hyperlaxity develop recurrent shoulder dislocation more easily. Many active military personnel who have congenital hyperlaxity may develop an increased sense of shoulder instability during training [1, 26]. With an increase in military training, the incidence of recurrent shoulder dislocation has increased significantly among young soldiers, and because some military units are located far away from hospitals, patients in the early stages of injury cannot receive effective specialized treatment. Thus, the incidence of bony Bankart lesions in military personnel has significantly increased due to long-term recurrent shoulder instability [3]. Recurrent shoulder instability is a complex disease, particularly in soldiers with joint hyperlaxity, which always requires surgical treatment [25].

For severe anterior inferior bony glenoid defects, it is difficult to restore the stability of the shoulder joint with simple soft tissue repair, which has a high postoperative recurrence rate [10, 11]. The current consensus is that bone augmentation surgery is recommended for recurrent dislocation with large glenoid bone defects [18]. The Latarjet surgical technique can effectively treat recurrent anterior shoulder instability; however, there are many related surgical complications [19, 24]. The Latarjet procedure can cause scapular dyskinesia, further increasing the complexity of revision surgery [21]. Because of the need to return to training, arthroscopic autologous iliac bone graft suspension fixation combined with the Remplissage technique was used to treat bony Bankart lesions of shoulder instability in patients with joint hyperlaxity. This study aimed to evaluate the short-term efficacy of this surgical approach in these patients. It was hypothesized that our technique would exhibit good clinical effects; if the glenoid bone defect is filled well and remodeled into the pear-shaped glenoid anatomy, it would be effective in preventing recurrent instability with joint hyperlaxity.

## Materials and methods

The study was approved by the institutional research ethics committee of the 940th Hospital of Logistics Support Force of PLA (NO. 2021KYLL114). Between March 2018 and September 2020, all patients who exhibited recurrent anterior shoulder instability combined with shoulder hyperlaxity and bone loss of the glenoid rim were included. Inclusion criteria were as follows: (1) patients experiencing recurrent anterior shoulder instability confirmed by history and physical examination; (2) Beighton joint range of motion score of ≥ 4; (3) “off-track” Hill–Sachs injury confirmed with 3-dimensional reconstructed computed tomography (3DCT), magnetic resonance imaging (MRI), and arthroscopy, with no bone graft required to fill the defect; and (4) postoperative follow-up of ≥ 1 year. The exclusion criteria were as follows: (1) revision surgery; (2) absence of shoulder hyperlaxity; (3) no substantial glenoid bone loss (< 15%); (4) previous shoulder operation; and (5) presence of neurological symptoms. In total, 22 patients (22 shoulders: 7 left and 15 right) met the inclusion criteria. The mean follow-up period was 19.3 ± 4.1 months. Patient demographics are listed in Table 1.

**Table 1:** Demographic data

Patients	Value (*N* = 22)
Number of dislocations, mean (range)	14.9 ± 6.3
Age at first dislocation, years	23.3 ± 4.1
Time from symptom onset to surgery, months	23.1 ± 13.0
Side, left/right, *n* (%)	7 (32%)/15 (68%)
Body mass index, kg/m^2^	23.6 ± 2.4
Bony defect, %, mean ± SD	18.8 ± 2.8

Data are reported as the mean ± SD unless otherwise indicated

*SD* standard deviation

### Surgical technique

**Fig. 1:**
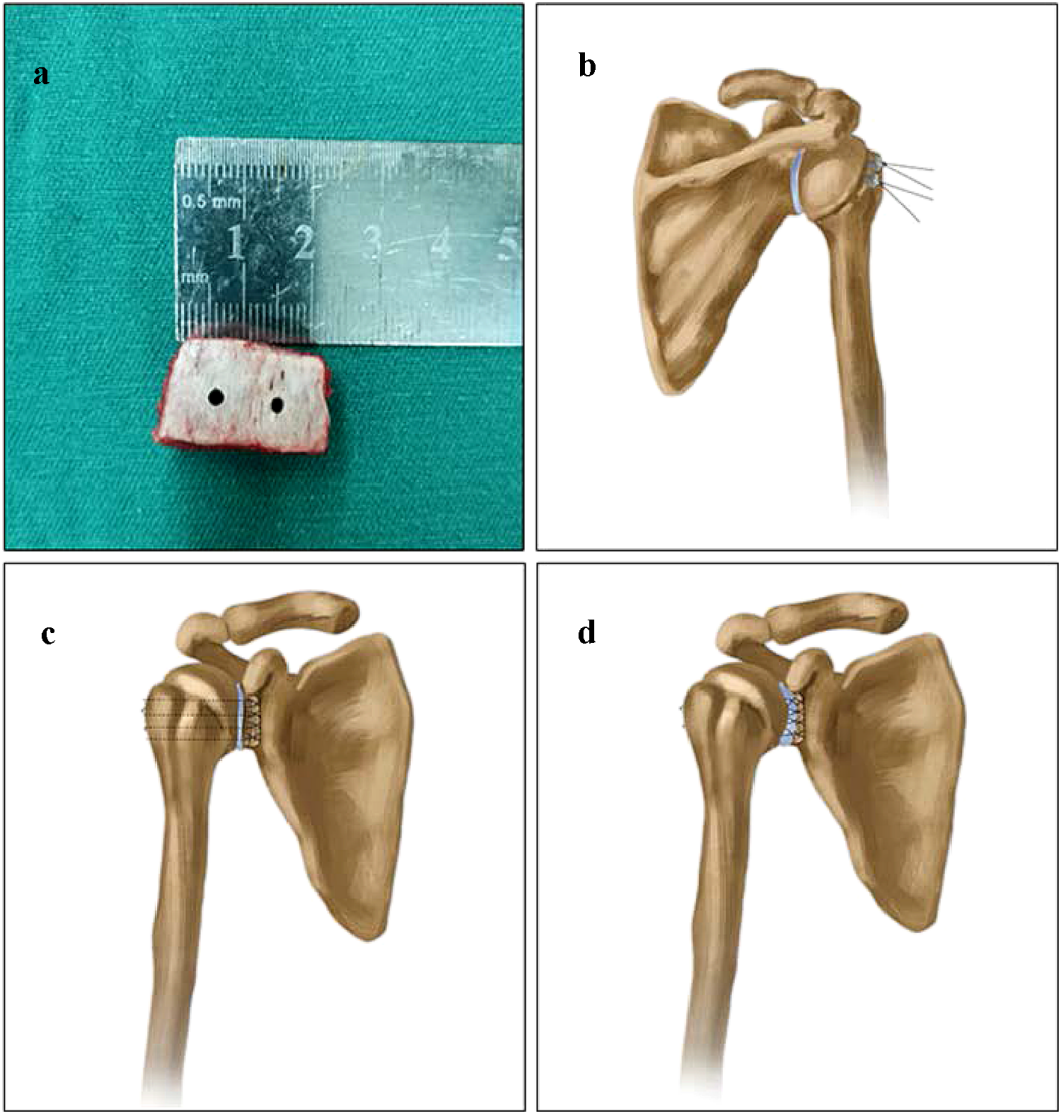
Schematic diagram of the surgical technique. **a** Preparation of the bicortical iliac crest graft, **b** Remplissage for the Hill–Sachs lesion, **c** placement of the graft in the anterior inferior labrum defect, and **d** anatomical glenoid reconstruction, together with suture and fixation of the capsule-labrum complex

After successful anesthesia, the patient was placed in the lateral decubitus position, and the affected arm was secured with an abduction traction frame. Arthroscopy was performed with the standard posterior, anterosuperior, and anteroinferior portals. The glenohumeral joint cavity structures were carefully inspected to assess the anterior inferior glenoid bone defect, which accounted for ≥ 15% of the glenoid area; the presence of Hill–Sachs lesions; whether bone grafting was needed; and whether the rotator cuff was complete. The region approximately 1 cm behind the anterior superior iliac spine was selected, and an oblique incision, approximately 3 cm backward, was made along the iliac spine. Based on the volume of the defect, the bicortical bone graft size was 2.0 × 1.0 × 1.0 cm, and two parallel 2 mm holes were drilled and prepared (Fig. 1a). With the scope in the posterolateral portal, one or two 4.5 mm suture anchors at the Hill–Sachs injury site were implanted, the penetrator reverse wire-passing device was used to thread the posterior joint capsule, and the anchor suture was led out without knotting (Fig. 1b). Observations through the anterior superior scope were as follows: the glenohumeral ligament-labral complex adhering to the scapular neck was released through the anterior inferior approach and the glenoid surface of the scapula was freshened and prepared. In the anterior inferior portal, 3.0 mm suture anchors were implanted at the 5:00 and 3:00 positions of the glenoid. The suture passer was used to shuttle the suture through the posterior arthroscopic cannula. One of the single wires of the 5-point and 3-point suture anchors was drawn out from the front portal and passed through the drilling holes of the bone graft. Then, the graft was suspended and fixed in the glenoid defect area by suture knotting. At the 5:00 anchor position, the suture passer was used to pass another anchor suture through the labral capsule complex (Fig. 1c). The sutures were then shuttled and tied arthroscopically in a simple or mattress fashion. Anchors at other sites were sequentially used to repair the labrum using the same technique (Fig. 1d). Viewing from the anterolateral superior portal, the posterior articular capsule was filled in the Hill–Sachs lesion by the Remplissage procedure.

### Postoperative rehabilitation

The patients were asked to use a shoulder brace for 6 weeks. Shoulder shrugging and passive “pendulum-like” movement were initiated immediately. After 6 weeks, the patients gradually started active shoulder exercise but avoided throwing movements for 3 months. Free functional exercise of the shoulder joint could be performed after 3 months. The patients were instructed to recheck the CT and a functional score of the shoulder joint at 6 weeks, 3 months, 6 months, and 1 year after the operation to adjust the treatment and rehabilitation training plan in time to ensure the maximum recovery of shoulder function and avoid the recurrence of injury. After the doctor’s evaluation and permission, unconstrained military training was allowed at 6 months post-operatively.

### Clinical evaluation

**Fig. 2:**
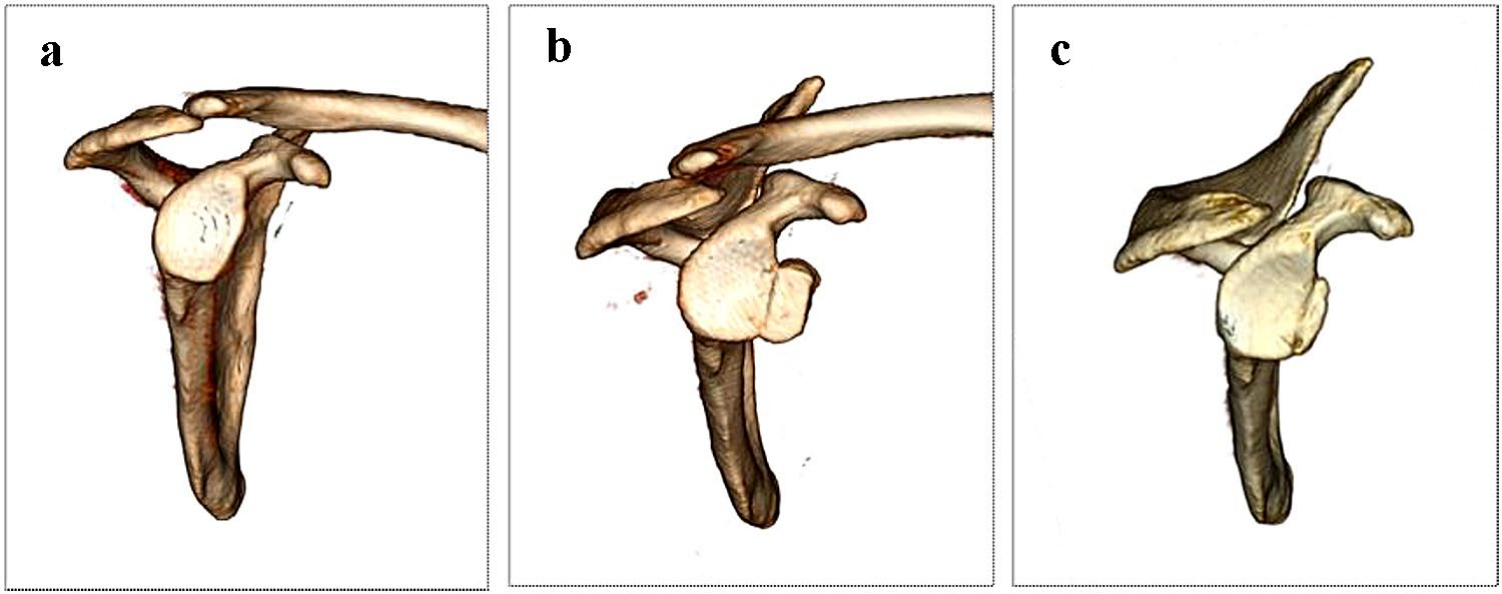
Remodeling of the graft and graft-glenoid interface. **a** Preoperatively, **b** immediately post-operatively, and **c** 1 year post-operatively. A small amount of bone resorption could be observed

Clinical examination was performed on admission and consisted of the University of California Los Angeles (UCLA) shoulder scale, Rowe score, visual analog scale (VAS) score, and Western Ontario Shoulder Instability Index (WOSI). Radiography and 3D CT were performed routinely for the preoperative and postoperative analysis of the glenoid and humeral bone defects (Fig. 2).

### Statistical analysis

Statistical analyses were performed using the SPSS software package (version 22.0; IBM, Armonk, NY, USA). A paired t test was used to compare the variations between the preoperative and postoperative results. For all analyses, significance was defined as *p* < 0.05. PASS 15.0 software was used to calculate the minimum sample size required for the study. The minimum sample size required for the VAS score was 12 participants, the minimum sample size required for the WOSI score was 6 participants, and the minimum sample size required for the UCLA score was 8 participants. Therefore, the number of samples collected in this study was 22, which met the minimum sample size requirements for all measures.

## Results

The characteristics of the study population are summarized in Table 1. The mean follow-up time was 19.3 ± 4.1 months. In total, 22 shoulders in 22 patients (1 female, 21 male) were included in the study. The mean patient age was 25.9 years. The mean defect area of the shoulder glenoid in all patients was 18.8 ± 2.8%, and the mean number of dislocations was 14.9 ± 6.3.

The VAS scores for pain throughout motion decreased from a mean of 5.9 ± 0.8 preoperatively to 2.2 ± 0.8 at the last follow-up (*p* < 0.001). The amelioration of pain throughout motion was statistically significant (Table 2). At the last follow-up, the Rowe and UCLA scores of the patients increased from 56.6 ± 4.7 and 13.4 ± 1.7 preoperatively to 89.4 ± 4.2 and 29.9 ± 2.1 post-operatively, respectively, which was statistically significant (*p* < 0.001; Table 2). Moreover, we found that the WOSI score decreased from 1492.7 ± 35.1 to 482.3 ± 46.2, which was statistically significant (*p* < 0.001). The glenoid surface area increased significantly, from 81.2 ± 2.8% before the operation to 119.5 ± 4.6% the day after the operation, and the glenoid defect area decreased significantly from 18.8 ± 2.8% before the operation to − 19.5 ± 4.6% the day after the operation. At 12 months after the operation, the surface area of the glenoid was reduced to 102.1 ± 6.2%, and the defect area of the glenoid was increased to − 2.1 ± 6.2%. Compared with the preoperative state, the measured glenoid area at the 12 months of postoperative follow-up was significantly increased, the glenoid defect area was significantly reduced, and no complete graft resorption was seen. No donor site complications were observed in this study. During this study, all patients had joint laxity, and the postoperative horizontal external rotation to 90° was not significantly affected. During this study, all patients remained on active duty and were able to recover pre-injury levels of normal daily life and training without dislocations.

**Table 2:** Preoperative and follow-up clinical outcomes

Parameter	Preoperative	Final follow-up	*p* value
VAS score	5.9 ± 0.8	2.2 ± 0.8	< 0.001
Rowe score	56.6 ± 4.7	89.4 ± 4.2	< 0.001
UCLA score	13.4 ± 1.7	29.9 ± 2.1	< 0.001
WOSI score	1492.7 ± 35.1	482.3 ± 46.2	< 0.001

Data are presented as the mean ± SD unless otherwise indicated

*SD* standard deviation, *UCLA* university california los angeles, *VAS* visual analog scale, *WOSI*, the western ontario shoulder instability index

## Discussion

The key finding of this study was that arthroscopic, autologous iliac crest bone grafting combined with Bankart repair and the Remplissage procedure provided excellent clinical and radiological results.

Recurrent shoulder instability is a complex disease with acquired joint hyperlaxity; the normal capsuloligamentous restraints of soldiers are overstretched due to repeated traction injury or repeated overuse during training and contact sports [16]. Due to excessive joint laxity, a dislocation/subluxation event in soldiers with anterior shoulder instability may occur easily and often, thereby aggravating the bone defect [17]. The treatment difficulty of the disease becomes more complex when recurrent shoulder dislocations and joint laxity exist in the same patient. Murphy et al. found that the failure rate of arthroscopic Bankart in the repair of anterior shoulder instability was greater than 30%, and the rate of recurrent instability was similar between patients with glenoid defects and patients with moderate bone loss and joint hyperlaxity [22]. Nakagawa et al. reported that 20.4% of athletes who engaged in contact or collision sports still had a recurrence of instability after straightforward arthroscopic Bankart repair [23]. When arthroscopic Bankart repair is used to treat recurrent anterior shoulder instability with bone defects, scholars believe that bone graft augmentation procedures should be strongly recommended [15].

The most commonly used glenoid reconstruction procedures embrace numerous coracoid displacement or bone-block procedures, similar to the Bristow-Latarjet, iliac crest autograft, or distal tibial allograft procedures [6, 31]. Bristow-Latarjet procedures release the pectoralis minor tendon, transpose the conjoined tendons, dam the subscapularis muscle, and alter the anatomy of the anterior shoulder. This procedure has a protracted learning curve and many complications, making any future revision surgery more difficult [12]. Griesser et al. analyzed 45 studies and reported that original or modified Bristow-Latarjet operations had a considerable risk of complications (30%), including recurrent dislocation (2.9%) and revision surgery (6.9%) [13]. Moroder et al. reported that the incidence of complications after free bone grafting ranged from 0 to 8.7%, and it can be used as an alternative to Latarjet surgery or as a revision treatment after failure of Latarjet surgery [20]. The clinical efficacy of open and arthroscopic Latarjet surgeries are comparable; a literature review noted that the complication rate of open Latarjet operation was approximately 15%, and the unplanned reoperation rate was 7% [12].

Burkhart et al. suggested an inverted pear-shaped glenoid as a risk factor for the failure of soft tissue Bankart surgery [8]. Open or arthroscopic iliac crest graft procedures are safe and effective with good clinical and radiologic results, and they can successfully reconstruct the pear-shaped glenoid anatomy [5, 7, 21, 30]. The surgical criteria for patients with recurrent anterior shoulder instability with bone defects include the restoration and reconstruction of the anatomical structure of the glenoid and glenohumeral stability. Warner et al. published his experience in the treatment of bony Bankart injury by reconstructing the glenoid joint with a tricortical iliac bone graft; the 33-month follow-up results showed that the bone graft was placed in the joint had a high healing rate and good stability. In this study, bicortical bone grafts were used, and clinical results showed that they also had a high healing rate [32].

The amount of glenoid bone loss is closely related to clinical efficacy and high recurrence rate. At present, there is still debate regarding the extent of glenoid bone loss that requires a bone graft augmentation procedure. Historically, 20–25% has been accepted as the indicator for bone reconstruction surgery. The study results of Shaha et al. show that when the bone loss exceeds 13.5%, patients are mostly unsatisfied with the clinical results of arthroscopic soft-tissue reconstruction [27, 28]. Calvo et al. found that when the articular glenoid defect was > 15%, it increased the danger of recurrent shoulder dislocation after Bankart repair [9]. Therefore, recurrent shoulder instability, glenoid bone loss of 15%, and excessive joint relaxation were considered to be the relative indicators of glenoid enlargement in this study. The patients included in this study were soldiers with high training intensity, a previous history of dislocation, and different degrees of glenoid bone defects accompanied by different degrees of joint laxity.

Considering the patients’ sports training needs and the expectations of recovering to the best state after the operation, glenoid bone defects and joint laxity needed to be treated simultaneously. To achieve this, a fully arthroscopic surgical operation combining autologous iliac crest bone grafting, Bankart repair, and Remplissage was used in this study. Autologous iliac crest bone grafting successfully reconstructed the pear-shaped anatomy of the shoulder glenoid and significantly restored glenohumeral stability. During the postoperative follow-up, no patients had redislocation, and all returned to pre-injury training levels. The Remplissage procedure could cause partial loss of shoulder external rotation function. There was no significant effect on external rotation ROM in the patients with joint relaxation symptoms included in this study. Additional analysis is still required to assess whether excessive joint laxity can compensate for the limited external rotation of the shoulder caused by surgery [17]. The all-arthroscopic modified Eden-Hybinette procedure was used to treat patients with anterior shoulder instability, and postoperative CT showed good graft bone healing with no cases of complete bone graft resorption [2, 30]. Zhao et al. improved iliac bone graft reconstruction by suspending the allogeneic iliac bone bicortical graft on the glenoid through anchor sutures in combination with Bankart repair, which resulted in 100% graft healing. This procedure restored the stability of the shoulder joint, but the article did not mention the absorption rate of the bicortical iliac allograft [33]. The postoperative follow-up showed that the incidence of bone resorption was low, but there was no graft control group in this study. The radiological outcomes of our study showed that the graft remodeled into the anatomic glenoid configuration and that only a small amount of bone resorption occurred; no patients had complete graft bone resorption. The rate of patient satisfaction was high.

This study has some limitations because of its retrospective design. First, the number of patients recruited for this study was small, and most patients were male soldiers. The applicability of the results to ordinary patients is limited. Second, this study did not include a control group, and the follow-up time was too short.

## Conclusion

All-arthroscopic autologous iliac crest bone grafting augmentation procedures, combined with Bankart repair and Remplissage, are currently a valuable method for the treatment of recurrent anterior shoulder dislocation in patients with glenoid defects and joint hyperlaxity.

## Supplementary Information

Below is the link to the electronic supplementary material.

Supplementary file1 (PDF 79 KB)
